# Salience-Based Edge Selection in Flicker and Binocular Color Vision

**DOI:** 10.1177/2041669520929047

**Published:** 2020-06-03

**Authors:** Stuart Anstis, Grace Hong, Alan Ho

**Affiliations:** Department of Psychology, University of California, San Diego; Department of Psychology, Ambrose University, Calgary, Alberta, Canada

**Keywords:** flicker, contrast, binocular, color

## Abstract

A test cross that flickers between light yellow and dark blue at 5 to 8Hz looks apparently yellow on a dark gray surround and apparently blue on a light gray surround (*flicker augmented contrast*). The achromatic surround cannot be inducing the perceived colors. Instead, the visual system *selects* the more salient apparent color with the higher Michelson contrast. The same is true for dichoptic vision. When one eye views a steady, light yellow cross and the other eye views a congruent steady dark blue cross, the binocular combination of colors looks apparently yellow on a dark gray surround and apparently blue on a light gray surround. Thus, when competing stimuli are distributed over time (flicker) or space (dichoptic vision), the visual system overweights the stimulus with the higher contrast. To see objects clearly, we accept the *best* view of any object and downplay inferior alternatives.

In simultaneous contrast (Heinemann, 1955), a gray test cross looks slightly darker when viewed against a white surround than against a black surround. We have previously reported a much stronger form of simultaneous contrast (Anstis & Ho, 1998): A cross that flickers between light yellow and dark blue at 5 to 8 Hz looks yellowish on a dark gray surround and bluish on a light gray surround. This is not caused by induction—an achromatic surround cannot induce colors (Anstis & Ho, 1998). Thus, when the cross alternates between a light and a dark hue, the visual system selects the more salient hue—the one with the higher Michelson contrast relative to the background (*flicker-augmented contrast*: Anstis, 2017; Anstis & Ho, 1998). In short, changing the surround luminance can completely change the appearance of a blue/yellow flickering cross.

We now extended our previous results by

1. *flickering* a dark blue/light yellow cross;
2. *binocular combination* of dichoptic crosses—one eye views a steady blue cross and the other eye views a congruent, steady yellow cross; and
3. *amalgamating* Conditions 1 and 2 by presenting a flickering blue/yellow cross to one eye and a yellow/blue cross, flickering in counterphase, to the other eye.

In all three conditions, the crosses were exposed on two different achromatic surrounds: a dark gray one, equiluminous with the dark blue cross, and light gray one, equiluminous with the light yellow cross. All three conditions can be demonstrated by viewing Movie 1 in three different ways:

1. *Flicker*. Run the movie. All crosses are flickering between identical blue and yellow. However, the upper crosses on the dark surround look light yellow, and the lower crosses on the light surround look dark blue (flicker augmented contrast).
2. *Dichoptic viewing.* Stop the movie and view the steady yellow crosses with one eye and the steady blue crosses with the other eye (cross your eyes to free fuse them). Result: The upper, binocularly combined cross on the dark surround looks light yellow, and the lower cross on the light surround looks dark blue.
3. *Amalgamation of 1 and 2*. Run the movie so that it flickers as in 1. Free-fuse by crossing your eyes as in 2. The flicker is in opposite phase in the two eyes, but the observer is not aware of this. Instead, as before, the upper cross looks yellow and the lower cross looks blue. This impression is if anything more stable than in 2.

We measured the perceived colors in Conditions 1 and 2. The two colors were always given different Michelson contrasts, but to reduce binocular rivalry (Alais & Blake, 2004), we never showed a spatial increment to one eye and a spatial decrement to the other.

Observers moved a mouse to adjust a colored bar, seen by both eyes, so that it varied between (say) dark blue through gray to light yellow. They adjusted this bar to give a best color match to the flickering or binocularly combined crosses. Three different color pairs were used, namely, dark blue versus light yellow (shown here), dark magenta versus light green, and dark red versus light cyan. Stimuli were generated in Adobe Director on a Macbook laptop computer and calibrated with a Minolta Chromameter II. Figure 1 shows the results.

**Figure 1.**
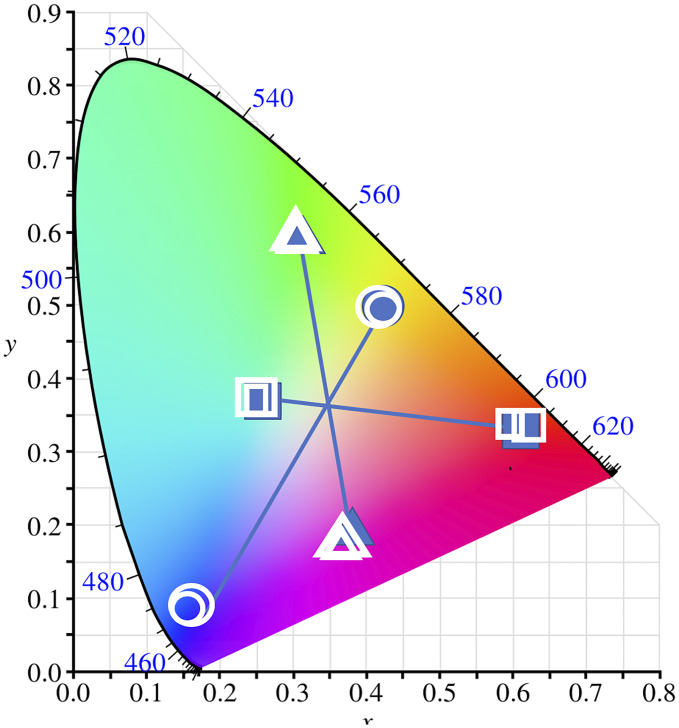
Filled Circles Show the Dark Blue and Yellow Crosses Used. Two open circles show the dark blue settings that observers matched to the appearance of the flickering (small circles) and dichoptic blue/yellow crosses (large circles) on the light gray surround. The other two open circles refer to the light yellow settings made for the same blue/yellow crosses on the dark gray surrounds. Thus, the cross color that matched the surround luminance was suppressed or ignored, and the opposite, complementary color dominated in winner-take-all fashion. Note that flickering and dichoptic presentations gave similar results. Similar conventions and results apply to the red/cyan (squares) and the magenta/green crosses (triangles; mean of two observers).

**Movie 1.**
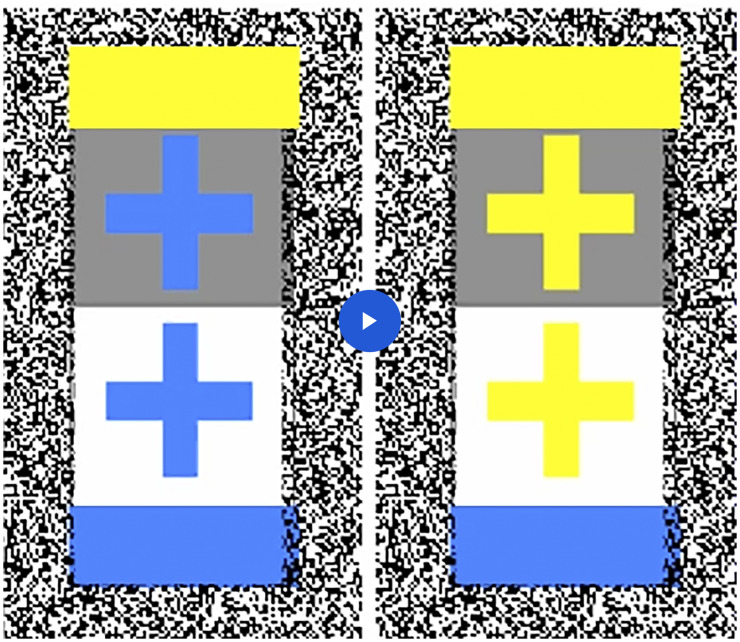
Flicker augmented contrast: Run the movie. Although all crosses flicker between dark blue and light yellow, the upper crosses on the dark surrounds look yellow and the lower crosses on the light surrounds look dark blue. Dichoptic viewing: Fuse the left and right displays by crossing your eyes. The upper, binocularly fused cross on the dark surround looks light yellow, and the lower fused cross on the light surround looks dark blue.

**Movie 2.**
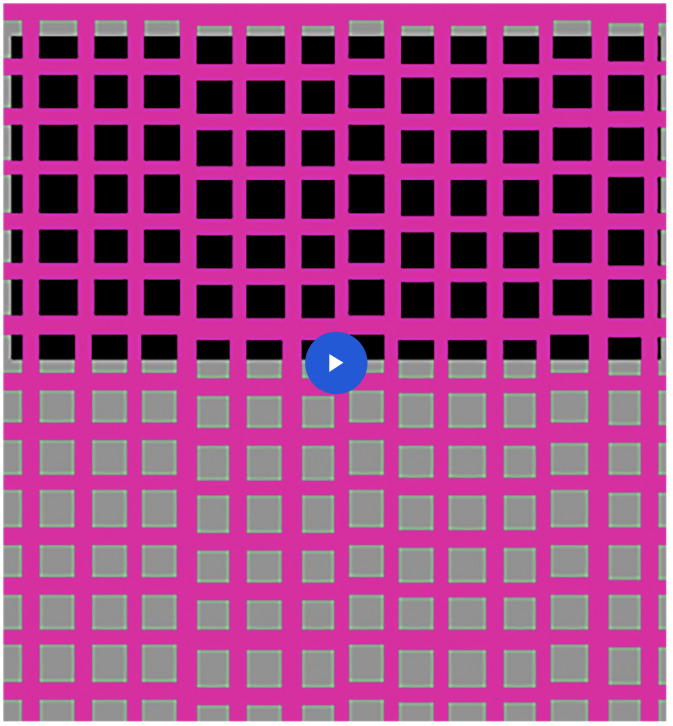
Although the entire grid flickers uniformly between green and magenta, it looks magenta in the upper half and green in the lower half.

In Movie 2, the entire grid flickers uniformly between green and magenta. The upper flickering background is black while the grid is magenta and equiluminous gray while it is green. So the magenta is higher in contrast than the green and the grid looks magenta. In the lower half, the opposite is true, so the grid looks green.

Not all dichoptic stimuli are like these. An orange and a lime disk, presented one to each eye, can combine into a single average yellow (Anstis & Rogers, 2012).

Comparable to our reports of monocular flicker versus binocular fusion, published reports describe two separate mechanisms that respond to (a) luminance versus contrast (Flynn & Shapiro, 2013), (b) monocular versus cyclopean brightness induction (Shevell et al., 1992), and (c) spatial integration at low color contrast versus edge responses at high color contrast (Shapley et al., 2019)
